# Viremia Associated with Fatal Outcomes in Ferrets Infected with Avian H5N1 Influenza Virus

**DOI:** 10.1371/journal.pone.0012099

**Published:** 2010-08-12

**Authors:** Xue Wang, Jiangqin Zhao, Shixing Tang, Zhiping Ye, Indira Hewlett

**Affiliations:** 1 Lab of Molecular Virology, Division of Emerging and Transfusion Transmitted Diseases, Food and Drug Administration, Bethesda, Maryland, United States of America; 2 Division of Viral Products, Center for Biologics Evaluation and Research, Food and Drug Administration, Bethesda, Maryland, United States of America; Institute of Infectious Disease and Molecular Medicine, South Africa

## Abstract

Avian H5N1 influenza viruses cause severe disease and high mortality in infected humans. However, tissue tropism and underlying pathogenesis of H5N1 virus infection in humans needs further investigation. The objective of this work was to study viremia, tissue tropism and disease pathogenesis of H5N1 virus infection in the susceptible ferret animal model. To evaluate the relationship of morbidity and mortality with virus loads, we performed studies in ferrets infected with the H5N1 strain A/VN/1203/04 to assess clinical signs after infection and virus load in lung, brain, ileum, nasal turbinate, nasal wash, and blood. We observed that H5N1 infection in ferrets is characterized by high virus load in the brain and and low levels in the ileum using real-time PCR. In addition, viral RNA was frequently detected in blood one or two days before death and associated with symptoms of diarrhea. Our observations further substantiate pathogenicity of H5N1 and further indicate that viremia may be a bio-marker for fatal outcomes in H5N1 infection.

## Introduction

In 1997, the avian influenza A virus subtype H5N1, which is a highly pathogenic H5N1 avian influenza initially confined to poultry, crossed the avian-human species barrier [1], [2], and has since emerged as a highly fatal infectious disease in the human population. To date, the World Health Organization has reported 467 laboratory-confirmed cases, 282 of which were fatal, resulting in a fatality rate of ∼60% (World Health Organization http://www.who.int/csr/disease/avian_influenza/country/cases_table_2009_12_30/en/index.html). Although it is more than 10 years since human H5N1 cases were reported, H5N1 influenza is poorly understood in terms of its pathology and pathogenesis. Only a limited number of reports describing pathological findings in human H5N1 cases have been published. Nevertheless, recent studies combined with early findings have gradually resulted in a better understanding of cell and organ pathology caused by H5N1, as well as viral tissue tropism.

The primary pathologic process that causes death is fulminant viral pneumonia [2]. High replication efficiency, broad tissue tropism and systemic replication seem to determine the pathogenicity of H5N1 viruses in animals [3]. The human isolate A/Vietnam/1203/04 (H5N1) was reported to be highly pathogenic and the severity of disease was associated with broad tissue tropism and high virus titers in multiple organs, including the brain [4]. In vitro and animal model studies indicate that high and disseminated viral replication is an important factor in disease pathogenesis [4]–[7]. Ferrets are an excellent mammalian animal model for studies of influenza virus pathogenicity and host immunity, and disease manifestations of influenza virus infection in ferrets closely resemble those in humans. To evaluate the relationship of morbidity and mortality with virus loads, we performed studies in ferrets infected with the H5N1 strain A/VN/1203/04 to assess clinical signs after infection and virus load in lung, brain, ileum, nasal turbinate, nasal wash, and blood.

## Results

### Disease Signs Caused by Human A/VN/1203/04

To evaluate virologic characteristics in susceptible ferrets, 24 ferrets (12/group) were challenged with either 1 FLD_50_ (50% ferret lethal dose) or 10 FLD_50_ of the H5N1 strain A/VN/1203/04 on Day 0, since these doses were known to cause symptomatic infection to varying degrees in the susceptible animal models. All ferrets were observed to be clinically normal on Day 0. Clinical signs of infection were initially observed on Day 2 postinfection with most animals exhibiting signs by Days 3 or 4 postinfection. Clinical signs observed in both groups included diarrhea, nasal discharge, hypoactivity and recumbency (Table 1). The number of ferrets that demonstrated clinical symptoms in the group that received the high dose (10 FLD_50_) of virus was higher than in the group receiving 1 FLD_50_. Greater mortality was observed in the high dose group, with three ferrets euthanized as scheduled two found dead on Day 4 and the remaining seven at Day 10 postinfection. In the low dose group, four ferrets were euthanized as scheduled on Day 4 postinfection, four ferrets were found dead between Days 6 and 8 and four ferrets survived until sacrifice on Day 11.

**Table 1:** Clinical observations in ferrets infected with H5N1 virus, A/VN/1203/04.

		Day Postinfection										
Group	Clinical Sign	1	2	3	4	5	6	7	8	9	10	11
10 FLD_50_	Diarrhea			2^a^	2	1	2		1	1		
	Discharge		5	7	9	7	7	3	2	1		
	Hypoactive			1	1							
	Recumbent						1		1			
	Found dead				2		1	3	1	1	1	
1 FLD_50_	Diarrhea			1	1	1	2	2	1		1	
	Discharge		3	4	9	7	6	5	4	2	2	
	Hypoactive			3	4	1	1	1	1			
	Recumbent						1	1	1			
	Found dead					1	1		1	1		

Note:

a) indicates number of surviving ferrets exhibiting clinical sign on the day postinfection.

**Figure 1:**
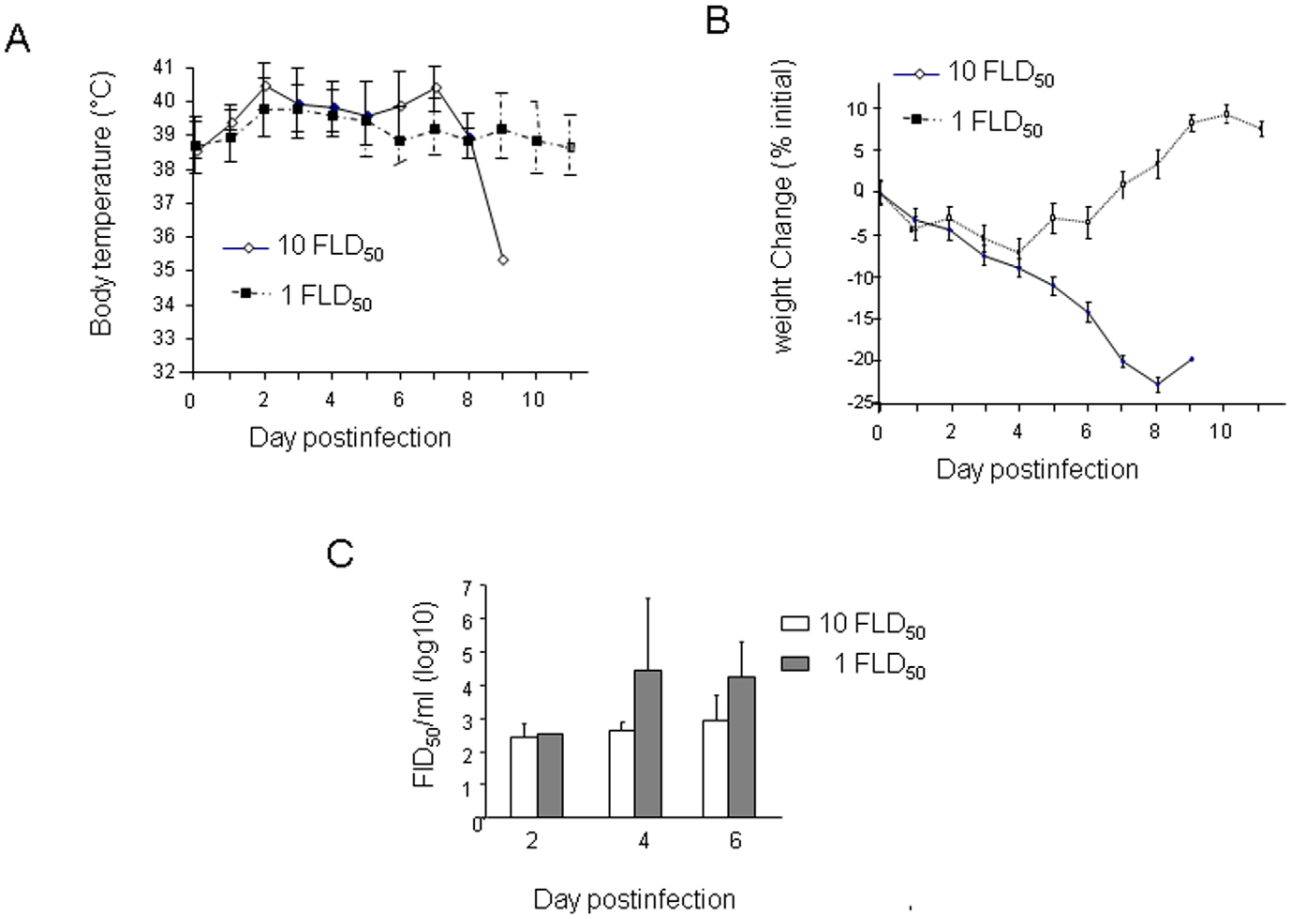
Clinical signs of infection with H5N1 virus A/VN/1203/04 in ferrets. (A) Changes in body temperatures of infected ferrets. Ferrets were inoculated with either 1 FLD_50_ or 10 FLD_50_ of the viruses, and body temperatures were monitored daily by the use of subcutaneous implantable temperature transponders for 11 days postinfection. Each data point represents the mean value ± SD for the surviving ferret(s). Note: only one animal remained alive in the 10 FLD_50_ group (one value) on Day 9 postinfection. (B). Changes in weights of ferrets infected with the viruses. The weights of ferrets were measured daily. The loss or gain of weight was calculated for each ferret as the percent change in the initial mean starting weight on day 0. Values are the averages ± SD for the ferret(s) alive for each group. (C) Virus titer in nasal wash with FLD_50_ assay. Nasal washes were performed on Day 2, Day 4, and Day 6 postinfection in selection of ferrets in both groups using FLD_50_ assay.

We examined all clinical symptoms during the course of infection to characterize the disease caused by H5N1 infection and to identify characteristics that explain the severity of infection. We observed high fevers (39.3∼41.5°C in the 10 FLD_50_ group and 38.3∼41.3°C in the 1 FLD_50_ group) on Day 2 or Day 7 postinfection (Fig. 1A), substantial weight loss (from 3% to 23% during Day 2 and Day 9 postinfection in the 10 FLD_50_ group; 6% by Day 4 postinfection in the 1 FLD_50_ group) (Fig. 1B). Diarrhea was one of the most frequently observed clinical symptoms, as all ferrets exhibited yellow diarrhea before death (Table 1).

There was a greater increase in weight loss and body temperatures for longer duration in the 10 FLD_50_ group compared to the 1 FLD_50_ group. In the latter group, ferrets regained body weight after Day 7 and their body temperature returned to normal after Day 8 post-infection, suggesting that some animals in this group recovered from the disease. Not surprisingly, the virus dose of 1 FLD_50_ was not sufficient to cause death in all ferrets, as the majority remained alive on Day 11 post-infection, the day of scheduled sacrifice. Using the FLD50 assay, we found that virus could be detected in the nasal washes more frequently in animals in the 10 FLD50 group compared with those in the 1 FLD50 group (Fig. 1C). Nasal wash samples had viral load detectable on Days 2 in both groups, and increased levels were observed at Days 4 and 6 using FLD_50_ assay (Fig. 1C).

### Virus Load in Organs

The severity of clinical signs and symptoms, including diarrhea, induced by infection with H5N1 indicated a rapid spread of the virus^4^ confirmed by virus detection in multiple organs, including the brain (Table 2), using the real-time PCR technique. There was a clear trend toward detection of higher titers of virus in the brain than in other organs, in both the 1 FLD_50_ and 10 FLD_50_ groups. The titers of virus in the lung were slightly lower than in the brain. Virus was also detected in the ileum (Table 2). These results indicate that disease severity or fatality caused by the virus was associated with broad issue tropism and high virus titers in multiple organs, particularly the brain.

**Table 2:** Viral load using TaqMan RT-PCR in ferrets infected with virus, A/VN/1203/04 (fg, mean±SD).

		Day Postinfection				
Group	Organ	4	5	6	7	8
	Brain	8.51×10^7^±1.16×10^8^ a		6.23×10^7^ b	3.88×10^7^±5.27×10^7^	3.80×10^7^±4.30×10^7^
10 FLD_50_	Lung	2.95×10^7^±1.71×10^6^		2.06×10^4^ b	1.10×10^6^±9.13×10^5^	8.51×10^6^±1.20×10^5^
	N.T.				1.88×10^4^±2.20×10^4^	3.40×10^4^±2.28×10^4^
	Ileum				4.44×10^3^±3.32×10^3^	485±337
	Brain		3.94×10^7^ b	7.76×10^7^ b		3.11×10^7^±4.59×10^6^
1 FLD_50_	Lung		2.70×10^3 ^ b	1.09×10^5^ b		3758±2822
	N.T.		9.33×10^4^ b	5.50×10^5^ b		8138±1094
	Ileum			4.36×10^3^ b		3.47±1.08

Note:

a) Known concentrations of A/VN/1203/04 viral RNA (serially diluted: 10^8^ to 10 fg) were used as templates and quantitative RT-PCR performed to generate a standard curve. 25∼30 mg of tissue from each organ was used for extraction of viral RNA and dissolved in 50 µl. 3 µl of the vRNA was used as template to perform real-time PCR. Each value represents the average concentration of six reactions based on the standard curve.

b) indicates value for 1 animal.

### Association of Viremia with Death of Ferrets

It has been previously reported that virus could be detected in blood in severely ill patients [5], [6]. We wished to further examine the relationship between viremia and fatal outcomes. To address this issue, we extracted nucleic acids from the blood of infected ferrets on Days 0, 1, 2, 4, and 6 postinfection with A/VN1203/04, and performed real-time RT-PCR (Table 3). We found that there was a clear tendency for virus to be detected in blood one or two days before the animal died (during the Day 4 and Day 8 period postinfection in the 10 FLD 50 group and between Day 5 and Day 6 postinfection in the 1 FLD50 group), with accompanying symptoms of diarrhea, suggesting that viremia could potentially be a predictable biomarker for animal death. We could not detect virus in the blood of two ferrets that died after Day 8 postinfection (the first on Day 9 postinfection, the second on Day 10 postinfection) of the 10 FLD 50 group, and two ferrets that died after Day 7 postinfection (the first on Day 8 postinfection, the second on Day 9 postinfection) in 1 FLD50 group. We also found a similar tendency in mice infected with the A/VN/1203/04 (H5N1) virus (data not shown). These data indicate that high titers of viremia are a strong predictor of death in the untreated host. For some animals that died later in infection there was no detectable viremia prior to death, under our experimental conditions.

**Table 3:** Detection of viremia by TaqMan RT-PCR and cases of death of ferrets infected with H5N1 virus, A/VN/1203/04.

Group	Animal code	Viremia a day postinfection	Diarrhea day postinfection	Died^ b^ day postinfection
	15	2	3	4
	23	2	3	4
	28	2		6
10 FLD_50_	17	6		7
	32	6	4	7
	33	6	6	7
	34	6		8
1 FLD_50_	29	2	3	5
	26	4		6

Note:

a) the day postinfection when virus was initially detected in the ferret blood sample.

b) the day postinfection when the animal was found dead.

## Discussion

H5N1 virus related influenza remains a relatively novel disease with poorly understood pathology and pathogenesis, and only a limited number of reports describing pathological findings in human H5N1 cases have been published [6]. Some studies have shown that H5N1 virus is found exclusively in the respiratory tract (mainly in the lung) [9], [10]. Other studies report the presence of H5N1 viruses in many extrapulmonary organs, such as intestine, liver, and brain [3]–[7]. Viral RNA has been detected in nasopharyngeal aspirates ranging from 1 day up to 15 days after disease onset [11], [12]. Viral replication appears to be prolonged in H5N1 influenza because viral loads when plotted against time did not show a clear decline in a large group of H5N1 patients [6]. Various animal studies indicate that aberrant production of proinflammatory cytokines and chemokines may play an important role in the pathogenesis of H5N1 influenza, which is consistent with abnormal regulation of cytokines and chemokines, including hemophagocytotic activity, that have been described in H5N1 autopsy cases [10], [12]–[14]. In many H5N1 patients elevated serum levels of proinflammatory cytokines and chemokines have been detected [6], [10], [12]. Apoptosis in alveolar cells and infiltrating leukocytes are prominent findings [15]. Lymphocyte depletion occurs in the spleen, lymph nodes, and tonsils; and reactive hemophagocytosis presumably result from host cytokine responses and viral infection.

Thus far, several hundred human infections with avian H5N1 viruses have been confirmed. The A/VN/1203/04 strain that we used in our study is a highly pathogenic isolate[4]. This viral strain caused viremia and was lethal to ferrets (Table 1). Viral sequences and antigens have been detected in lymphocytes in lymph node tissue, as well as in Hofbauer cells (macrophages of the placenta), Kupffer cells (macrophages of the liver), and mononuclear cells in the intestinal mucosa [15], [16]. Recently, it has been reported that viral RNA was found in the blood of humans with fatal outcomes while no viral RNA could be detected in the blood of surviving H5N1-infected individuals [6]. This is consistent with the findings we report here in ferrets. Accordingly, extra-pulmonary dissemination may be the result of viremia or of infected immune cells transporting virus to other organs, although this remains to be demonstrated. Viremia was accompanied by low peripheral blood T-lymphocyte counts and high chemokine and cytokine levels in humans infected with H5N1 viruses [6], suggesting that high viral load and the resulting acute inflammatory responses have interconnected roles in influenza H5N1 pathogenesis. Taken together, these findings highlight the need for further studies to examine the relevance of viremia in H5N1 pathogenesis and virus transmission.

## Materials and Methods

### Virus

Avian H5N1 strain A/VN/1203/04 was obtained from the Centers for Disease Control and Prevention (Atlanta, GA) (Lot Number: E3/E2 1/18/07) and amplified in viable 10–11 day old embryonated hen's eggs (S&G Poultry, Clanton, AL). The virus was maintained at −80°C until use in the study.

### Determination of Viral titers

50 µl of allantoic fluid were harvested from euthanized hen's eggs and added to a microtiter plate. 50 µl of 0.5% turkey (tRBC) were then added to all wells and plates were incubated for 30 minutes at room temperature. Plates were read for agglutination or non-agglutination. The 50% endpoint was determined by the method of Reed and Muench^17^ from virus dilutions testing positive for hemagglutinin activity in Turkey Red Blood Cells (tRBC). Data were expressed as 50% egg infectious dose (EID_50_) per milliliter.

### Inoculation of ferrets

24 adult male ferrets (Triple F Farms, Sayre, PA) (The Southern Research IACUC has approved the animal care and use proposal (ACUP) #08-10-074B by IACUC Chairman, Larry Bowen) that were 6∼7 months of age and were seronegative for representative currently circulating human influenza A strains prior to shipment were used for the study. Ferrets were lightly anesthetized with a solution of ketamine/xylazine/atropine formulated to provide doses of 25 mg/kg ketamine, 1.7 mg/kg xylazine, and 0.05 mg/kg of atropine to each animal. The animals were inoculated intranasally with one ml of virus, approximately 500 µl to each nare; 12 of ferrets with 1×50% of ferret lethal dose (FLD50), which equals to a concentration of 10×10^8.5^ (EID_50_/ml) viruses and others with ten times of FLD_50_/ml viruses. Clinical signs of infection, weight, and temperatures were recorded daily. Nasal wash samples were collected from all ferrets on Days 0 (approximately 12 hours post-dose), 1, 2, 4, 6, and 8 for viral load determination. A total of 0.5 ml from each animal were collected and used to determine the viral load using the EID_50_ assay. Nasal washes were performed by slowly dripping 0.5 ml of sterile solution of 1% v/v BSA, 100 U/ml penicillin, 100 µg/ml streptomycin, and 50 µg/ml gentamicin in Dulbecco's Phosphate-Buffered Saline into each nare, while allowing the animal to sneeze and/or attempt to blow the solution out of the nostril into a sterile Petri dish. Nasal wash samples were made into aliquots in cryovials and placed on dry ice immediately after collection. Samples were stored at −80°C until used.

### Tissue collection

A representative section of the left caudal lobe of the lung, nasal turbinate, spleen, brain, and ileum tissue samples were collected from each euthanized or deceased animal for viral load determination. Samples were snapped frozen in liquid nitrogen and stored at −80°C until analyzed for viral load determination. On Days 0, 1, 2, 4, and 6 postinfection, blood samples were collected from surviving ferrets, and stored at −80°C until used.

### Titration of virus in nasal washes

Nasal washes were thawed and cleared by centrifugation. The resulting supernatants were serially diluted (log_10_) in DPBS (Dulbecco's Phosphate-Buffered Salines with antibiotics, 100 U/ml Penicillin, l00 µg/ml Streptomycin and 50 µg/ml Gentamicin) (Invitrogen, Carlsbad, CA). The 50% endpoint was determined by the method of Reed and Muench [8] from egg dilutions testing positive for hemagglutinin activity in tRBC. Virus titers were expressed as EID50/ml. The limit of detection was 1×10^0.5^ EID_50_/100 µl since the initial sample dilution was 1∶10.

### Real-time PCR

Quantitative real-time RT-PCR was also used for detection of virus load in the brain, lung, nasal turbinates, ileum, and blood. Nucleic acids were isolated by using the QIAamp Viral RNA Mini Kit (Valencia, CA 91355) according to the manufacturer's protocol. We designed a set of primers and probes for the matrix gene, M, of the avian H5N1 influenza A virus, according to GenBank database. The forward primer was 5′-CGTCAGGCCCCCTCAAA-3′, and the reverse primer was 5′-GGTGTTCTTTCCTGCAAAGA-3′. The TaqMan probe was oligonucleotide 5′-TCAAGTTTCTGTGCGATCT-3′, coupled with a reporter dye [6-carboxy fluorescein] (FAM) at the 5′ end, a non-fluorescent quencher and a minor groove binder (MGB), that served as a Tm enhancer, at the 3′ end. The nucleic acids were amplified and detected in an automated TaqMan 7500 Analyzer by using QuantiTect™ Probe RT-PCR kit (Qiagen Inc., Valencia, CA). The 25-µl PCR mixture consisted of 100 nM primers and 100 nM probe. Following three thermal steps at 55°C for 5 min, at 50°C for 30 min and at 95°C for 10 min, 45 cycles of two-step PCR at 95°C for 15 s and at 60°C for 1 min were performed. The limit of detection was 1 fg of virus RNA per reaction with the TaqMan assay since the initial sample dilution was 1∶10.
